# Hedgehog interacting protein (HHIP) represses airway remodeling and metabolic reprogramming in COPD-derived airway smooth muscle cells

**DOI:** 10.1038/s41598-021-88434-x

**Published:** 2021-04-27

**Authors:** Yan Li, Li Zhang, Francesca Polverino, Feng Guo, Yuan Hao, Taotao Lao, Shuang Xu, Lijia Li, Betty Pham, Caroline A. Owen, Xiaobo Zhou

**Affiliations:** 1 School of Medicine, Cheeloo College of Medicine, Shandong University, Jinan, 250012 Shandong Province China; 2 Channing Division of Network Medicine, Brigham and Women’s Hospital and Harvard Medical School, Boston, MA 02115 USA; 3 Department of Integrated Traditional Chinese and Western Medicine, West China Hospital, Sichuan University, Chengdu, 610041 China; 4 Asthma and Airway Disease Research Center, University of Arizona, Medicine, Tucson, AZ 85724 USA; 5 Center for Immunology and Inflammatory Diseases, Massachusetts General Hospital, Boston, MA 02129 USA; 6 Division of Pulmonary and Critical Care Medicine, Department of Medicine, Brigham and Women’s Hospital and Harvard Medical School, Boston, MA 02115 USA; 7 Present Address: Center for Reproductive Medicine, Cheeloo College of Medicine, Shandong University, Jinan, 250012 Shandong Province China

**Keywords:** Functional genomics, Cell growth

## Abstract

Although HHIP locus has been consistently associated with the susceptibility to COPD including airway remodeling and emphysema in genome-wide association studies, the molecular mechanism underlying this genetic association remains incompletely understood. By utilizing *Hhip*^+*/-*^ mice and primary human airway smooth muscle cells (ASMCs), here we aim to determine whether HHIP haploinsufficiency increases airway smooth muscle mass by reprogramming glucose metabolism, thus contributing to airway remodeling in COPD pathogenesis. The mRNA levels of HHIP were compared in normal and COPD-derived ASMCs. Mitochondrial oxygen consumption rate and lactate levels in the medium were measured in COPD-derived ASMCs with or without HHIP overexpression as readouts of glucose oxidative phosphorylation and aerobic glycolysis rates. The proliferation rate was measured in healthy and COPD-derived ASMCs treated with or without 2-DG. Smooth muscle mass around airways was measured by immunofluorescence staining for α-smooth muscle actin (α-SMA) in lung sections from *Hhip*^+*/-*^ mice and their wild type littermates, *Hhip*^+*/*+^ mice. Airway remodeling was assessed in *Hhip*^+*/-*^ and *Hhip*^+*/-*^ mice exposed to 6 months of cigarette smoke. Our results show HHIP inhibited aerobic glycolysis and represses cell proliferation in COPD-derived ASMCs. Notably, knockdown of HHIP in normal ASMCs increased PKM2 activity. Importantly, *Hhip*^+*/-*^ mice demonstrated increased airway remodeling and increased intensity of α-SMA staining around airways compared to *Hhip*^+*/*+^ mice. In conclusion, our findings suggest that HHIP represses aerobic glycolysis and ASMCs hyperplasia, which may contribute to the increased airway remodeling in *Hhip*^+*/-*^ mice.

## Introduction

Chronic obstructive pulmonary disease (COPD) ranks as the third leading cause of global death^1^. COPD is characterized as emphysematous destruction of the alveoli and thickening of the airway wall caused by cellular and structural changes, which is referred to as airway remodeling. Many pathological changes including hyperplasia and hypertrophy of airway smooth muscle cells (ASMCs), infiltration of immune cells, epithelial cell hyperplasia, goblet cell metaplasia, and subepithelial fibrosis contribute to airway remodeling, which promotes irreversible lung airflow obstruction^2^ during COPD pathogenesis^3^. Increased ASMC mass around airways, characteristic of airway remodeling, is inversely correlated with lung function (FEV_1%_ predicted)^4^ and positively related to COPD severity^5^. In contrast to severe asthmatics with increased airway smooth muscle mass in large airways, COPD patients tend to have increased airway smooth muscle mass mainly in small airways^6^.

Although genome-wide association studies (GWAS) have identified the 4q31 locus^7–9^ nearby *HHIP* (hedgehog interacting protein) gene as one of the most replicated loci in COPD, including both emphysema^10,11^ and airway remodeling^12^, as well as pulmonary function in general populations^2,13–15^, the molecular mechanisms underlying this genetic association remain largely unknown. Previously, we have reported that the COPD risk allele at the HHIP GWAS locus is associated with reduced distal enhancer activity for HHIP and reduced expression of HHIP in lungs^16^. *Hhip* knockout mice (*Hhip*^*-/-*^) died immediately after birth due to defective lung branching morphogenesis^17^, but *Hhip* heterozygous mice (*Hhip*^+*/-*^) were previously well characterized for their transcriptomic, metabolic and molecular features in lungs related to COPD^18,19^. *Hhip*^+*/-*^ mice also recapitulate multiple human COPD pathological features, including cigarette smoke-^20^ and age-^21^ related emphysema. However, whether and how Hhip regulates airway remodeling, especially airway smooth muscle cellular change in *Hhip*^+*/-*^ mice, remains to be determined.

There is mounting evidence that dysregulated cellular metabolism occurs in various lung diseases, including pulmonary fibrosis and acute lung injury^22,23^. However, relatively few studies have been performed to investigate metabolic changes in COPD pathogenesis. Previously, we showed another COPD GWAS gene, *FAM13A*, promoted fatty acid-oxidation in airway epithelial cells that might contribute to cigarette smoke-induced cell death during emphysema development^24^, suggesting the importance of genetic factors in regulating cellular metabolism during COPD development. Recently, metabolic reprogramming of glucose metabolism towards aerobic glycolysis was reported in COPD-derived ASMCs^25^. A previous study has demonstrated COPD-derived ASMCs exhibit a higher degree of cellular proliferation than normal ASMCs in response to FBS stimulation^26^. Moreover, decreased basal mitochondrial oxygen consumption rate (OCR) due to impaired mitochondrial function^27^ and increased metabolic shift to aerobic glycolysis (Warburg effects) have also been revealed in COPD-derived ASMCs compared to normal ASMCs^25^. However, the correlation between this glucose metabolic shift from oxidative phosphorylation (OXPHOS) to aerobic glycolysis and increased proliferation in COPD-derived ASMCs have not been assessed. Moreover, our group has demonstrated the HHIP alleviates oxidative stress possibly by modulating mitochondrial function^21^, but the possible roles of HHIP in regulating oxidative phosphorylation in mitochondrial metabolism have not been studied yet. Based on previous findings, the current study investigates whether *HHIP*, the COPD GWAS gene, regulates glucose metabolic reprogramming and the biological consequence of such metabolic changes in ASMCs.

Herein, we hypothesize that HHIP protects ASMCs from metabolic reprogramming towards aerobic glycolysis, preventing airway remodeling by inhibiting ASMC hyperplasia in COPD pathogenesis. Our results show that Hhip haploinsufficiency promotes airway remodeling in *Hhip*^+*/-*^ mice, as indicated by increased airway smooth muscle mass around airways. Moreover, lower HHIP expression in COPD ASMCs are associated with increased aerobic glycolysis-mediated cell hyperproliferation.

## Materials and methods

### Animals

*Hhip*^+*/−*^ mice in C57BL/6 J background were described previously^20^. All mice were housed in the animal facility of Harvard Medical School with a 12 h light/12 h dark cycle. This study was performed in strict accordance with the recommendations in the Guide for the Care and Use of Laboratory Animals^28^ of the National Institutes of Health. All animal studies were approved by the Institutional Animal Care and Use Committee, Brigham and Women's Hospital.

### Cell culture

Human primary ASMCs derived from healthy donors (#CC-2576, N = 4, passage 2) and COPD patients (#00,195,274, N = 4, passage 2) were purchased from Lonza (Walkersville, MD). As shown in Supplemental Table 1, the age, gender, smoking, and alcohol use characteristics of healthy donors and COPD patients are comparable. The passages of normal and COPD-derived ASMCs are also comparable for each experiment. ASMCs were cultured in SmGM-2 BulletKit medium (CC-3182, Lonza). Cells were grown to sub-confluence before experiments. Cells were cultured at 37 °C in a humidified 5% CO_2_/air atmosphere, and the medium was changed every other day.

### Detection of β–gal activity: X-gal staining

X-gal staining was performed to indicate the expression of Hhip in murine lungs. *Hhip*^+*/-*^ mice were generated with insertion of the bacterial *lacZ* gene to replace the start codon and the rest of the downstream sequences in the first exon of the murine *Hhip* gene; thus the expression of LacZ gene indicated by X-gal staining reflects endogenous expression pattern of *Hhip* in murine lungs^29^. The detailed methods were described previously^21^. Briefly, murine lungs were inflated with X-gal solution at room temperature for 5 h, followed by fixation overnight in fresh 4% paraformaldehyde before sectioning.

### Immunofluorescence staining

Lung sections from *Hhip*^+*/*+^ and *Hhip*^+*/-*^ mice at the age of 8 months (N = 4–5 for each group) were stained with anti-α-SMA antibody (#M085129-2, Dako, Santa Clara, CA) at 1:400 dilution. Airways around 300–600 μm in diameter were chosen for quantification of α-SMA intensity surrounding airways by pixel intensity measurements using ImageJ software. The intensities (size of α-SMA positive staining area) were then normalized to the area of airways.

### Trichrome staining

Paraffin-embedded lung sections from *Hhip*^+*/*+^ and *Hhip*^+*/-*^ mice at the age of 8 months with or without CS exposure for 6 months were used for Masson's trichrome staining. To quantify small airway remodeling in CS-exposed mice, ImageJ software (Version 1.8.0, NIH) was utilized as previously reported^30^ to quantify the size of the deposition area of ECM proteins around the small airways (airways having a mean diameter between 300 and 699 μm were included). The positive areas for trichrome staining were then normalized to corresponding small airway area and statistically analyzed. The normal distribution of data was examined using Shapiro–Wilk normality test. Since these data were normally distributed, one-way ANOVA for multiple comparisons were then used to compare the effects of genotype and treatment.

### Cigarette smoke (CS) exposure in mice

Female *Hhip*^+*/-*^ and littermate mice (approximately 10 weeks old)^20^ were exposed to mixed main-stream and side-stream CS from 3R4F Kentucky Research cigarettes for 5 days/week in Teague TE 10z Chambers (Total Suspended Particulates approximately 100 to 200 mg/m^3^ and CO levels around 6 ppm). As a control, mice were exposed to filtered air for the same duration. At the end of the 6-month exposure period, lung mechanics were then measured in mice exposed to CS or air.

### Measurement of lung mechanics

Respiratory mechanics including respiratory system resistance and newtonian resistance in unchallenged *Hhip*^+*/*+^ (N = 14) and *Hhip*^+*/-*^ (N = 19) mice were measured as previously described^21^. The reduced expression of hhip in *Hhip*^+*/-*^ mice has also been confirmed previously^20^.

### Pyruvate kinase activity measurement

The pyruvate kinase activity was measured by the colorimetric method based on the manufacturer's instructions (Pyruvate Kinase Activity Assay Kit, Sigma). Briefly, 1X10^6^ cells were homogenized with pyruvate kinase assay buffer followed by absorbance measurement at a wavelength of 570 nm. The results were calculated based on the standard curve generated using the same kit.

### Small interfering RNA (siRNA) and plasmid transfection

For siRNA knockdown experiments, according to the manufacturer's instructions (Dharmacon, Lafayette, CO), ASMC cells were transfected with 20 nM ON-TARGETplus SMARTpool siRNA (si-HHIP, si-PKM2) or 20 nM siCONTROL NON-TARGETINGpool siRNA (Dharmacon, Lafayette, CO) for 48 h using Opti-MEM and Lipofectamine RNAiMAX. For overexpression experiments, CMV-HA-FLAG vector or HHIP-HA-FLAG plasmid at the concentration of 0.5 μg/ml were transfected into ASMCs using Lipofectamine 3000 Reagent (Invitrogen, Thermo Fisher Scientific) followed by culture medium change after 8 h of transfection. RT-qPCR or Western blot was performed to assess transfection efficiency.

#### Western blotting

Cells were washed with cold phosphate-buffered saline (PBS) twice and lysed in ice-cold RIPA buffer (Cell Signaling Technology, Danvers, MA) with added protease inhibitor cocktail (Bimake, Houston, TX), phosphatase inhibitor cocktail (Bimake, Houston, TX) and 1 mM PMSF (Sigma-Aldrich, Burlington, MA). Equal amounts of protein were separated by 4–15% gradient SDS-PAGE (Bio-Rad, Hercules, CA) and electrotransferred to polyvinylidene fluoride membrane. Membranes were blocked with TBST containing 5% (wt/vol) non-fat dry milk for 1 h and incubated overnight at 4 °C with primary antibodies diluted in TBST with 5% (wt/vol) non-fat dried milk and 0.1% (vol/vol) Tween-20. After incubation with appropriate horseradish peroxidase-conjugated anti-mouse or anti-rabbit IgG (GE Healthcare, Burlington, MA) secondary antibody for 1 h at room temperature followed by thorough washing in TBST, the membrane was developed for immunoreactive bands detection by G:BOX image developing system (Syngene Frederick, MD) with an enhanced chemiluminescent or supersignal west femto chemiluminescent substrates (Thermo Fisher Scientific, Grand Island, NY). Blot images in TIFF format were captured by G:Box image software and then ranged in Powerpoint software without any inappropriate contrast modification. Band densities were quantified by ImageJ (NIH) software with α-tubulin as the internal loading control for normalization.

#### Reverse transcription quantitative real-time PCR (RT-qPCR)

Total cellular RNA was extracted with RNeasy (Qiagen, Germantown, MD) kit according to the manufacturer's instructions. cDNA was synthesized using high-capacity cDNA reverse transcription kit (Applied Biosystems, Grand Island, NY). RT-qPCR was performed using Taqman gene expression master mix and QuantStudio 12 K Flex (Thermo Fisher Scientific, Grand Island, NY). At least three independent experiments were performed, and each sample was assayed in triplicates to determine the mRNA levels measured by QuantStudio 12 K Flex software v1.2.2 with comparative Ct method with GAPDH as the reference gene and using the formula 2^-ΔΔCt^.

#### Measurements of mitochondrial respiration

Extracellular Flux Analyzer (Seahorse Bioscience, Santa Clara, CA) was used to measure the oxygen consumption rate (OCR), an indicator of mitochondrial respiration, in primary human lung ASMCs in a 24-well plate. Briefly, cells were seeded directly into XF24 plates, and mitochondrial respiration was measured in Agilent Seahorse XF Base Medium (Agilent Technologies, Santa Clara, CA) supplemented with D-glucose (25 mM) and pyruvate (10 mM). ASMCs were seeded at a density of 3 × 10^4^ cells/well, and OCR was measured using the Seahorse XF Cell Mito Stress Test program. During the Seahorse assay, oligomycin (2 mM), carbonyl cyanide-4-(trifluoromethoxy) phenylhydrazone (2 mM) and antimycin/rotenone (1 mM of each) were sequentially added into each well for measurements of basal respiration, ATP production, maximum respiration, and proton leak (complex I driven), respectively.

#### Measurement of secreted lactate levels in cell culture media

Cells were seeded into a 96-well plate at the density of 3 × 10^3^ cells/well. The culture medium was then collected 24 h after the seeding of cells. The lactate levels in the medium were measured by the Lactate Colorimetric Assay Kit II (#K627-100, BioVision, Milpitas, CA), and lactate levels were then normalized to DNA content measured in each well at the time of collection.

#### Cell growth measurement

At the beginning of the cell growth experiment (Day 0), cells with various treatments were seeded into six copies of 96-well plates at a density of 3 × 10^3^ cells/well in triplicate wells. After cells were attached to the well (around 4–6 h following seeding), one of six plates was collected after supernatant removal, and stored with attached ASMCs at − 80 °C (Day 0, which indicates the seeding day). The remaining five 96-well plates were then collected subsequentially in the following 7–8 days using the same method and stored at -80 °C. After collecting all six plates, DNA contents of each well from six 96-well plates were measured using the CyQUANT NF Cell Proliferation Assay Kit (#C35006, Invitrogen, Grand Island, NY). DNA content readings from day 1 to day 7 or 8 were then normalized to day 0 readings (the first plate collected) for statistical quantification to reflect the relative cell number fold changes as several days of culture. All primary human ASMCs from Lonza were cultured in SmGM-2 BulletKit medium (CC-3182, Lonza), which contains 1.0 g/L glucose, 5% FBS, and growth supplements including human epidermal growth factor, Insulin, human fibroblast growth factor-beta (FGF-2) as well as gentamicin/amphotericin-B. Cultures were incubated at 37 °C in a humidified 5% CO_2_/air atmosphere, and the medium was changed every other day.

#### Antibodies, reagents and plasmid construction

HHIP was cloned into the pC-FLAG-HA CMV vector with BamHI and EcoRV restriction sites to generate HA-FLAG-HHIP constructs followed by sequencing confirmation as described previously^21^. Primary antibodies used in this study include anti-α-tubulin (#ab40742, Abcam, Cambridge, MA), anti-Hemagglutinin (HA) (C29F4) rabbit mAb (#3724S, CST, Danvers, MA), anti-PKM2 (#ab137791, Abcam, Cambridge, MA). Pyruvate Kinase Assay Kit (#ab83432) was purchased from Abcam.

#### Statistical analysis

All statistical analyses were performed using PRISM software (GraphPad Software, Inc. San Diego, CA). Unpaired Student's t-test were used for comparisons of two groups with normally distributed data, respectively. The normal distribution of data including α-SMA staining and trichrome staining in *Hhip*^+*/*+^ and *Hhip*^+*/-*^ mice with or without CS was confirmed using Shapiro–Wilk normality test. We also performed one-way ANOVA for multiple comparisons of means to compare the effect of genotype and treatment under each given condition. Means were considered significantly different if *P* < 0.05.

## Results

### A metabolic shift to aerobic glycolysis in COPD-derived ASMCs is essential for COPD-derived ASMC hyper-proliferation

**Figure 1:**
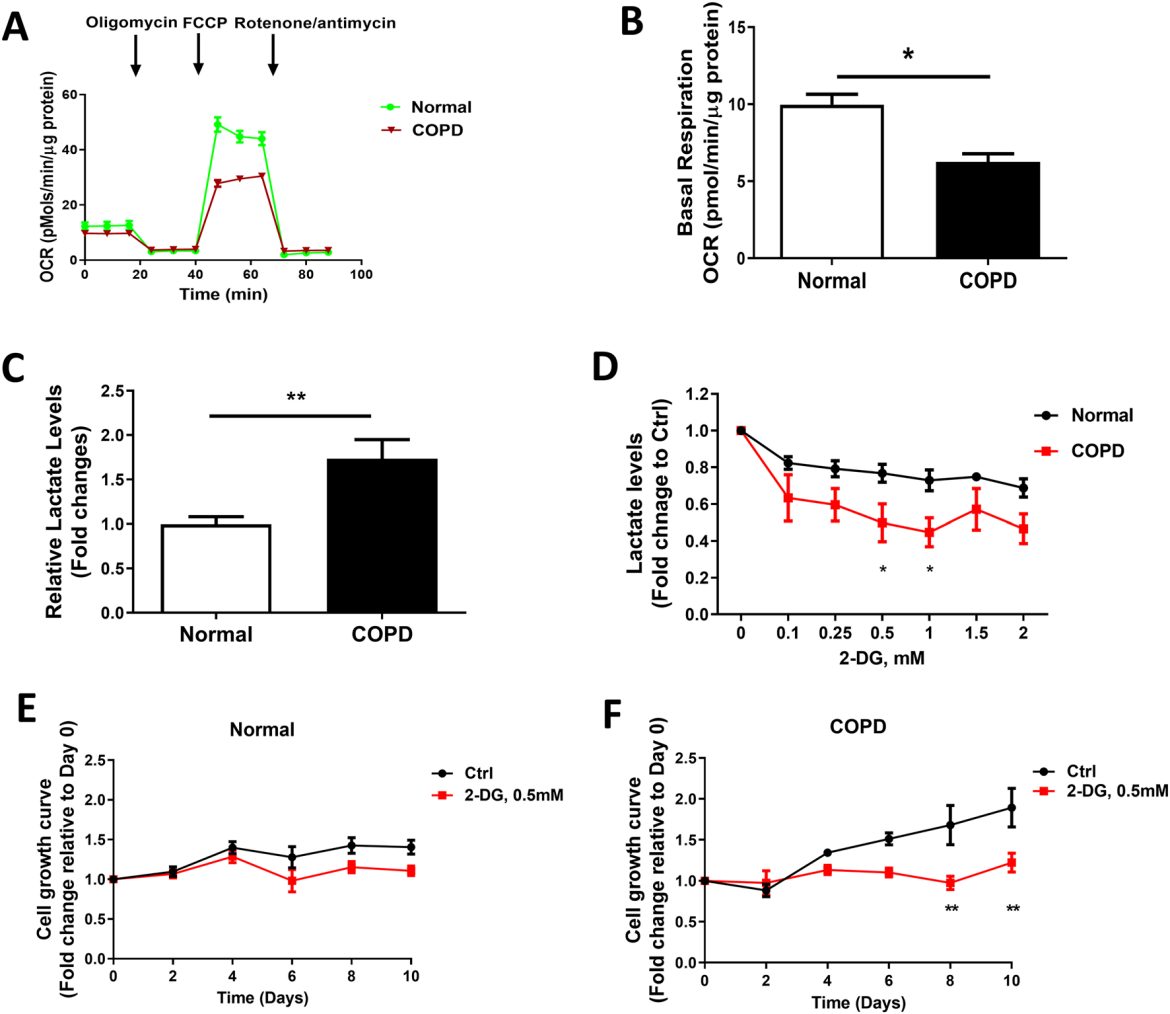
COPD-derived ASMCs demonstrated decreased oxygen consumption rate (OCR), increased lactate production, and increased sensitivity to glycolysis inhibition as well as its associated growth inhibition. (**A**,**B**) Oxygen consumption rate (OCR) in ASMCs from a healthy individual and COPD patient measured by Seahorse Mitostress assay. (**C**) Relative levels of lactate in the culture medium from healthy ASMCs (N = 4) and COPD-derived ASMCs (N = 3) normalized to DNA content in corresponding wells. (**D**) Relative levels of lactate were measured in the culture medium of normal ASMCs (N = 4) and COPD-derived ASMCs (N = 3) treated with 2-Deoxy-D-glucose (2-DG) at various concentrations (0-2 mM) for 24 h. Relative cell growth curve of normal ASMCs (N = 4) (**E**) or COPD-derived ASMCs (N = 3) (**F**) with or without 2-DG treatment (0.5 mM). *P < 0.05, **P < 0.01 by unpaired t-test. Means ± SEM from biological or technical repeats shown for each group.

To examine the metabolic reprogramming from oxidative phosphorylation (OXPHOS) to aerobic glycolysis in COPD-derived ASMCs, seahorse mitostress assay was performed, demonstrating decreased basal oxygen consumption rate (OCR) in COPD-derived ASMCs compared to normal ASMCs (Fig. 1A and B). Furthermore, lactate levels, indicating aerobic glycolysis, were increased in COPD-derived ASMCs compared to normal ASMCs (Fig. 1C). These results suggested the reprogramming of glucose metabolism from OXPHOS to aerobic glycolysis (Warburg effects) in COPD-derived ASMCs. To determine whether COPD-derived ASMCs rely on glycolysis to provide sufficient energy for their proliferation, we applied glycolysis inhibitor 2-Deoxy-D-glucose (2-DG), a competitive glucose surrogate, in both COPD-derived and healthy ASMCs. Interestingly, COPD-derived ASMCs demonstrated a more significant reduction of glycolysis upon 2-DG treatment compared to normal ASMCs (Fig. 1D). A previous study has identified the hyperproliferation of COPD-derived ASMCs in response to FBS^26^, our results embedded in Fig. 1E and F also support the hyperproliferation rate of COPD-derived ASMCs compared to normal ASMCs with the presence of FBS. In addition, COPD-derived ASMCs showed decreased cell proliferation rates when treated with 2-DG (0.5 mM), in contrast to normal ASMCs (Fig. 1E and F). Furthermore, 2-DG treatment has minimal effects on OCR in COPD-derived ASMCs (Supplemental Fig. 1A and B), suggesting reduced cell proflieration upon 2-DG treatment likely result from reduced glycolysis (Fig. 1D). Our results indicate that glycolysis inhibition is more effective for decreasing the proliferation of COPD-derived ASMCs which have higher levels of active glycolysis compared to healthy ASMCs (Fig. 1E and F).

### HHIP attenuated metabolic reprogramming towards aerobic glycolysis in COPD-derived ASMCs

**Figure 2:**
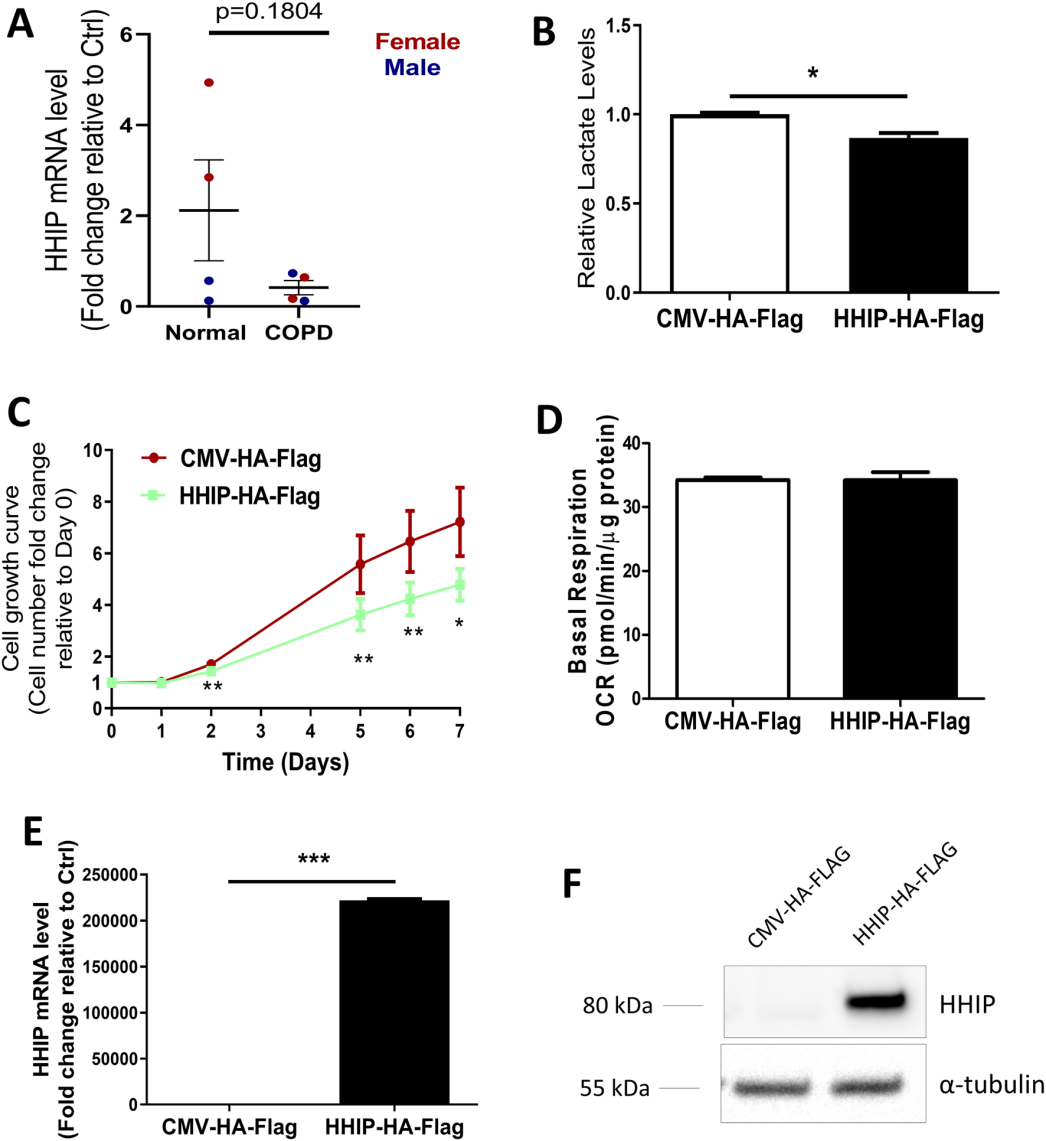
HHIP repressed proliferation and glycolytic metabolic reprogramming of COPD-derived ASMCs. (**A**) The HHIP mRNA levels were measured in ASMCs from healthy individuals (N = 4) and COPD patients (N = 4) by RT-qPCR. Red dots indicate data from female subjects, and blue dots indicate data from male subjects. (**B**–**F**) COPD-derived ASMCs were transfected with either HHIP overexpressing plasmid or empty vector, followed by measurements of (**B**) Lactate levels, (**C**) proliferation rates, (**D**) basal OCR, (**E**) HHIP mRNA levels, and (**F**) HHIP protein levels. *P < 0.05, **P < 0.01 by unpaired t-test. Means ± SEM from 3–4 biological and technical repeats are shown for each group.

We previously found decreased expression of HHIP in human COPD lungs (18) in COPD-derived ASMCs compared and healthy ASMCs (Fig. 2A), significantly reduced expression of HHIP mRNA levels was noted in COPD-derived ASMCs compared to normal ASMCs from female subjects, but not in male subjects). Current HHIP antibodies were unable to detect endogenous HHIP, future experiments are warranted to measure HHIP in normal and COPD-derived ASMCs at protein levels. Given important roles of glycolysis in COPD ASMCs, we examined possible regulation of glycolysis by HHIP. Indeed, overexpression of HHIP in COPD-derived ASMCs led to modest but significant reductions of lactate levels (Fig. 2B), decreased cell proliferation (Fig. 2C), without significant changes of mitochondrial OCR (Fig. 2D), suggesting HHIP overexpression altered aerobic glycolysis and cell proliferation rate without affecting mitochondrial OXPHOS in AMSCs. The overexpression efficiency was confirmed by RT-qPCR (Fig. 2E) and Western blot (Fig. 2F). These results suggest that HHIP may inhibit COPD-derived ASMC hyperproliferation and attenuated the elevated aerobic glycolysis detected in these cells.

### HHIP inhibits the activity of PKM2 that promotes aerobic glycolysis in COPD-derived ASMCs

**Figure 3:**
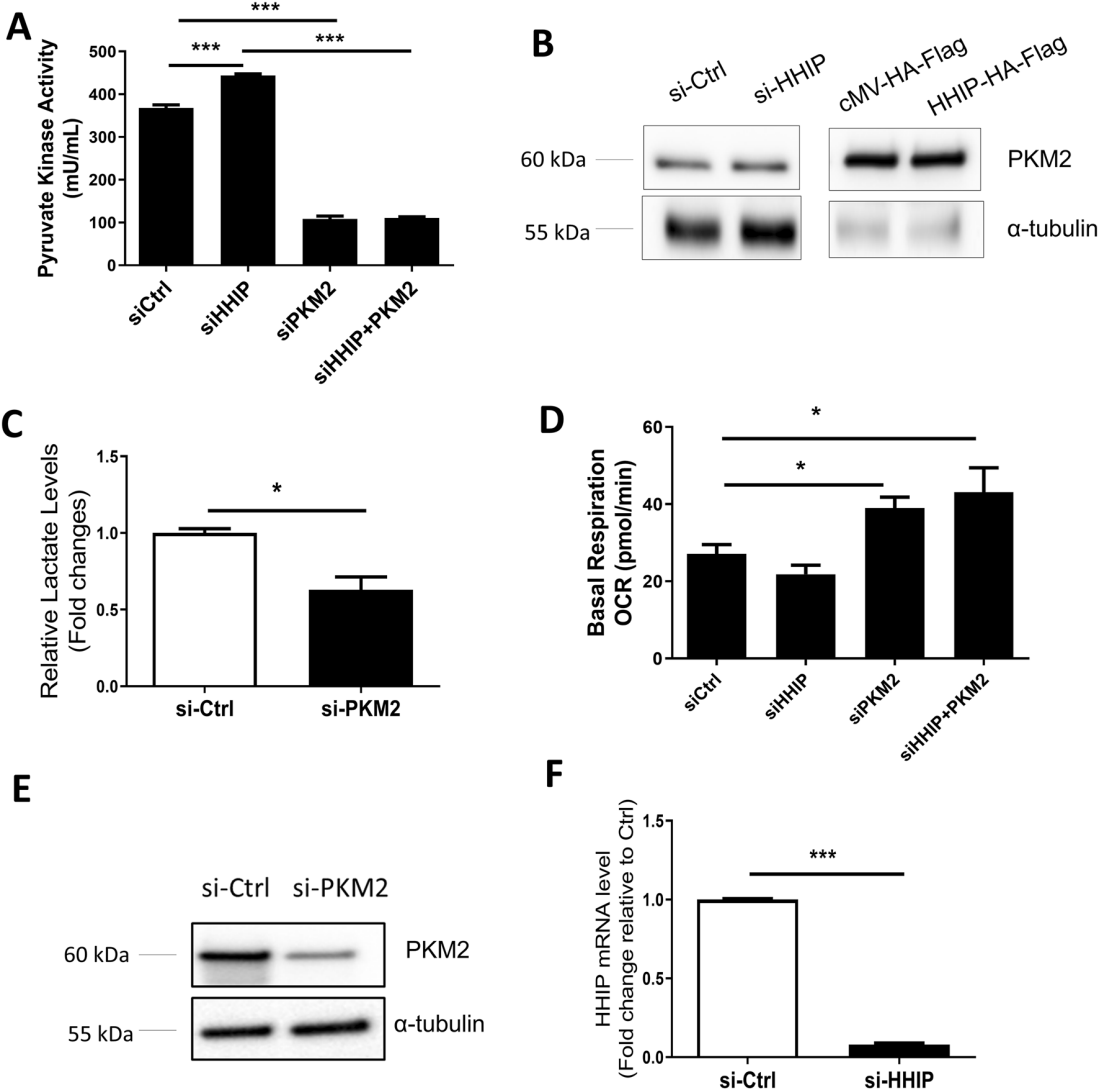
PKM2 activity is essential for metabolic reprogramming towards aerobic glycolysis in COPD-derived ASMCs. (**A**) Pyruvate kinase activity was measured in normal ASMCs with si-RNA mediated HHIP and/or PKM2 knockdown with normalization to protein concentration. (**B**) The PKM2 expression levels were examined by Western blot after HHIP knockdown in normal ASMCs (Left panel) or HHIP overexpression in COPD-derived ASMCs (Right panel). (**C**) Lactate levels in the culture medium were measured in COPD-derived ASMCs transfected with si-Ctrl or si-PKM2. (**D**) Basal OCR was measured in COPD-derived ASMCs transfected with si-Ctrl or siHHIP and/or si-PKM2. (**E**)The knockdown efficiency of si-PKM2 was shown by Western blotting. si-Ctrl: control siRNA; si-PKM2, PKM2 specific siRNA pool. (**F**) The knockdown efficiency of si-HHIP was shown by RT-PCR results. *P < 0.05, **P < 0.01 by unpaired t-test. Means ± SEM from 3 independent experiments shown for each group.

To identify potential mechanisms by which HHIP represses the metabolic reprogramming in COPD-derived ASMCs, we measured the activity of PKM2, a critical rate-limiting enzyme for glycolysis in muscle cells. The knockdown of HHIP significantly increased the pyruvate kinase activity in normal ASMCs, which were abolished by transfection of si-PKM2 (Fig. 3A). However, the PKM2 protein levels were not altered by either HHIP overexpression or knockdown in healthy or COPD-derived ASMCs, respectively (Fig. 3B). To determine the role of PKM2 in regulating glycolysis of COPD-derived ASMC, si-RNA mediated PKM2 knockdown with or without HHIP knockdown was applied to COPD-derived ASMCs followed by measurements of lactate levels, basal OCR, and pyruvate kinase activity. Our data showed that knockdown of PKM2 significantly decreased lactate levels (Fig. 3C) and increased basal OCR (Fig. 3D). The knockdown efficiency of PKM2 was confirmed by Western blot (Fig. 3E). The knockdown efficiency of HHIP was confirmed by Taqman RT-qPCR (Fig. 3F). In summary, HHIP may repress airway remodeling by inhibiting glycolytic reprogramming-induced ASMC hyperproliferation, which could be partly through inhibiting PKM2 activity, thus preventing airway remodeling in COPD pathogenesis.

### Hhip is expressed in murine lung ASMCs

**Figure 4:**
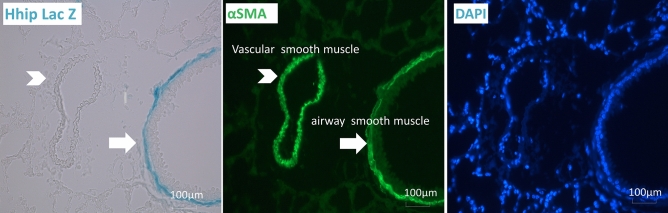
Hhip is expressed in ASMCs (airway lung) as indicated by LacZ staining in *Hhip*^+*/-*^ mice. A representative image of histology of lung sections from unchallenged *Hhip*^+*/-*^ mice at 2 months of age. LacZ staining (left panel), α-smooth muscle actin staining (middle panel), and DAPI staining (right panel) are demonstrated. Scale bars, 100 μm. Arrows indicate the localization of Hhip in the lungs. Arrowheads indicate vascular smooth muscle cells.

A recently published single-cell RNA-Seq data in murine lungs has indicated the expression of HHIP is enriched in ASMCs^31^. Consistent with this finding, our results demonstrate positive β-gal signals, indicative of Hhip expression in *Hhip*^+*/-*^ mice, are present in cells with positive α-smooth muscle actin staining (α-SMA) in the proximal airway, suggesting that Hhip is expressed in ASMCs or myofibroblasts. Interestingly, the location of HHIP is specifically in airway smooth muscle cells but not vascular smooth muscle cells (Fig. 4), suggesting exclusive localization of Hhip in ASMCs.

### Exacerbated airway remodeling and increased respiratory resistance in ***Hhip***^+***/-***^ mice

**Figure 5:**
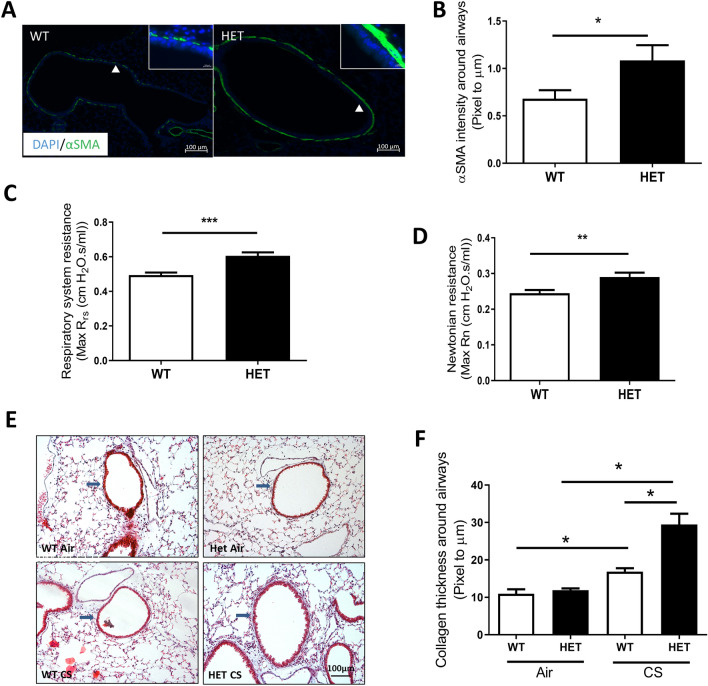
Immunostaining and lung function measurement in *Hhip*^+*/-*^ (HET) and *Hhip*^+*/*+^ (WT) mice. (**A**) Representative immunofluorescence staining of α-smooth muscle actin (SMA) in lung sections from *Hhip*^+*/-*^ (HET) vs. *Hhip*^+*/*+^ (WT) at the age of 8 months (Scale bars, 100 μm). Green color, α-SMA staining; Blue color, DAPI staining. Magnified figures from areas indicated by arrowheads are shown at the top right corner (Scale bars, 10 μm). (**B**) Quantification on α-SMA staining intensity around airways (N = 10–12 airways) in mice from each group (4–5 mice per group). Airways around 300–699 μm in diameter were chosen for quantification. Mean ± SEM are from airways (N = 10–12 airways) in each group. Lung mechanics including (**C**) Respiratory system resistance (Rrs) and (**D**) Newtonian resistance were measured in WT (*Hhip*^+*/*+^) and HET (*Hhip*^+*/-*^*)* mice at the age of 8 months (N = 14 vs. 19 mice). *P < 0.05 by Mann–Whitney test. (**E**) Trichrome staining in lung sections from 8-month-old female *Hhip*^+*/*+^ and *Hhip*^+*/−*^ mice with or without 6 months of CS exposure. (**F**) Quantification of Trichrome staining in murine airways (N = 10–12 airways) from each group (5–7 mice *per* group). Mean ± SEM are from airways (N = 10–12 airways) *per* group. Scale bars, 100 μm. WT, *Hhip*^+*/*+^ mice; HET: *Hhip*^+*/-*^ mice; CS, cigarette smoke. *P < 0.05 by one-way ANOVA. (Diameter of the airways assessed is 300–600 μm).

To determine the effects of Hhip haploinsufficiency on ASMCs function in vivo, we performed α-SMA immunofluorescence (IF) staining in lung sections from unchallenged *Hhip*^+*/-*^ and *Hhip*^+*/*+^ mice at 8 months of age preceding spontaneous emphysema development in *Hhip*^+*/-*^ mice at 10 months of age^21^. Our results showed Hhip haploinsufficiency spontaneously led to significantly increased α-SMA staining intensity around the airways (Fig. 5A and B). Additionally, compared to *Hhip*^+*/*+^ mice, *Hhip*^+*/−*^ mice showed significantly increased respiratory system resistance (Max Rrs) and newtonian resistance (Max Rn) (Fig. 5C and D) which are indicatives of greater total and central airway resistance respectively, consistent with the greater airway smooth muscle mass in *Hhip*^+*/-*^ mice. Moreover, collagen thickness around airways, as indicated by Trichrome staining^32^, were increased in CS-exposed *Hhip*^+*/-*^ mice compared to CS-exposed *Hhip*^+*/*+^ mice (Fig. 5E and F), suggesting haploinsufficiency of Hhip led to increased collagen deposition around airways in mice exposed chronically to CS. These results provide additional evidence supporting an inhibitory effect of Hhip on airway remodeling.

## Discussion

As the third leading cause of global death, COPD susceptibility is influenced by both environmental factors and genetic determinants. Airway remodeling, including an increased amount of ASMCs around airways, is inversely correlated with lung function^4^ and positively related to COPD severity^5^. Although GWAS has identified that chromosome 4q31 locus is significantly associated with COPD susceptibility and lung function^7–9^, the role of HHIP in airway remodeling is unknown^20,21^. In murine models, we demonstrate that *Hhip*^+*/-*^ mice spontaneously develop airway remodeling at 8 months of age as indicated by increased α-SMA intensity around airways and increased airway resistance compared to WT littermates. In primary human ASMCs, we found that metabolic reprogramming from OXPHOS to aerobic glycolysis contributes to cell proliferation in COPD-derived ASMCs^25–27^. Our previously published paper has shown that the Hhip mRNA levels in murine lungs were not altered by CS exposure for six months^20^. Here the expression of HHIP showed a trend toward reduction in female COPD-derived ASMCs. However, the interpretation of this finding requires caution due to the limited subjects used in our study. Importantly, HHIP was shown to attenuate aerobic glycolysis and cell hyperproliferation in COPD-derived ASMCs.

Airway remodeling in COPD, including the structural changes of airways, could be induced by increased levels of growth factors and cytokines^33,34^. Histological examination of airways and high-resolution CT are usually used to assess and quantify the extent of airway remodeling, which contributes to irreversible airway narrowing in COPD pathogenesis^35^. Growth factors such as TGF-β initiate airway remodeling by stimulating smooth muscle cell proliferation, activating fibroblasts to produce collagen and other ECM components, leading epithelial cells to transdifferentiate into myofibroblasts expressing mesenchymal markers such as α-SMA and vimentin^36^. Previously, increased airway smooth muscle mass has been shown in COPD patients at Gold stage III and IV compared to healthy individuals^5^. Importantly, studies suggest that increased muscle mass likely contributes to increased lung resistance observed in COPD^37^. Since airway remodeling in COPD leads to lung function reduction that is not fully reversed by corticosteroids and β-agonists^38^, the development of novel therapeutic interventions based on a better mechanistic understanding of airway remodeling is urgently needed. Recent reports on the genetic determinant of airway remodeling on COPD illuminate novel insights into the development of airway remodeling. Genetic variants nearby HHIP and MECR (mitochondrial trans-2-enoyl-CoA reductase) were previously implicated in small airway obstruction even in non-smokers^12^. Interestingly, *MECR* is a crucial gene involved in the last step of mitochondrial fatty acid synthesis. This finding further suggests that metabolic changes in airways may genetically determine airway obstruction.

Cellular metabolic homeostasis is essential for maintaining cell function, including proliferation and differentiation^39^. Glucose present in normal cells usually undergoes oxidative phosphorylation to generate ATP under normoxic conditions. However, hyperproliferative cells, such as cancer cells, rely on aerobic glycolysis rather than glucose OXPHOS to maintain a high cellular proliferation rate despite sufficient oxygen supply. Such metabolic reprogramming is known as the Warburg effect^40^. Though aerobic glycolysis is relatively less efficient in producing adenosine 5′-triphosphate (ATP), it facilitates the uptake and incorporation of nutrients into the biomass needed for new cells^41^. Recent studies reported imbalanced cellular bioenergetics in COPD patients. For example, disrupted lipid metabolism was detected in serum and sputum samples from human COPD patients^42,43^, as well as in CS-induced murine emphysema models^44^ consistent with dysregulated metabolism occurring during COPD development^42^. In murine models, CS increased fatty acid oxidation in airway epithelial cells^45^. In healthy human subjects, long-term smoking led to altered metabolites in airway basal cells, as assessed by global metabolic profiling^46^. Recently, a Warburg-like effect was reported in COPD-derived ASMCs and was speculated to contribute to the airway remodeling occurring in COPD^12^. In particular, Teperino et al*.* have reported that activation of the hedgehog pathway promoted Warburg-like metabolism in muscle^47^, highlighting the essential role of hedgehog pathway in regulating glycolytic metabolism. However, inhibitory effects of Hhip on the Hedgehog pathway relies on the competitive binding with three types of Hedgehog ligands present in the culture condition. Therefore, whether Hedgehog pathway is involved in the regulation of Hhip in the glycolysis in airway smooth muscle cells needs further investigation. Herein, using murine genetic models targeting the most replicated COPD GWAS gene-*HHIP*, we demonstrated increased airway remodeling in *Hhip*^+*/-*^ mice associated with increases in total and central airway resistance increase. Importantly, the metabolic shift of the Warburg-like effect from oxidative phosphorylation to aerobic glycolysis in COPD-derived ASMCs is ameliorated by HHIP, providing the first evidence for contributions of HHIP to the repression of metabolic reprogramming towards aerobic glycolysis. Notably, although the Warburg effect was observed in COPD-derived ASMCs as indicated by increased lactate levels and decreased OCR compared to normal ASMCs, transient overexpression of HHIP attenuates increased lactate levels modestly without influencing OCR in COPD-derived ASMCs, indicating incomplete rescue of metabolic reprogramming in COPD-derived ASMCs by resuming HHIP levels, possibly due to insufficient genetic changes over a short period against decades of accumulated metabolic.

Interestingly, expression of HHIP showed a reduced trend in COPD-derived ASMCs in female subjects. However, a larger sample size of ASMCs from healthy and COPD subjects from both genders is needed in future studies to explore sex-genetic interaction in determining HHIP expression and/or lung function since the number of primary cells used for these in vitro experiments are limited. Besides, whether differential hormone levels in male and female patients play essential roles in people carrying susceptibility genetic loci during COPD pathogenesis needs further investigation. Of note, transient restoration of HHIP levels by overexpression in COPD-derived ASMCs is insufficient to reprogram these cells back to the normal-like metabolic condition of ASMCs, suggesting chronic metabolic shift in COPD-derived ASMCs may result from multiple genetic changes combined over time. However, we indeed found female *Hhip*^+*/-*^ mice showed increased airway obstruction and airway remodeling compared to age-matched WT mice, supporting the importance of HHIP to maintain airway homeostasis. Future evaluations on the stress-induced airway obstruction such as under methacholine challenge in *Hhip*^+*/-*^ mice may facilitate a better understanding of the regulation of HHIP on airway function.

One possible mechanism by which HHIP modulates glycolysis in ASMCs is through the rate-limiting glycolytic enzyme-PKM2, which catalyzes the final step in glycolysis to produce pyruvate. PKM2 is selectively expressed in highly proliferative cells^48^. It has been shown that PKM2 activity is necessary for sustaining proliferative signaling in cancer cells^49^. By interacting with HIF-1α, PKM2 promotes the Warburg effect in macrophages^50,51^. In our current study, we found that HHIP reduces PKM2 activity in ASMCs, which is essential for metabolic reprogramming from OXPHOS towards aerobic glycolysis in COPD-derived ASMCs. Furthermore, we demonstrate that expression levels of PKM2 was not regulated by Hhip. Future studies are warranted to determine how HHIP regulates pyruvate kinase activity, possibly by influencing PKM2 phosphorylation, or interacting with PKM2, or affecting the conformational changes of PKM2, i.e., the transition between tetramer and dimer related to its activity in the lung cancer cell lines. Furthermore, whether PKM2 contributes to CS-induced airway remodeling in *Hhip*^+*/-*^ mice requires additional investigations on compound genetic deficient mice in the CS exposure model. Other than PKM2, other molecular mediators responsible for the effect of HHIP on metabolic reprogramming in ASMCs, such as p-AKT^52^, p-AMPK^47^, mTOR, and HIF-1α^53^ require additional studies. In the future, to comprehensively evaluate the effect of Hhip in modulating airway resistance, provocation/induced bronchoconstriction experiments, such as methacholine responsiveness assessment, are needed in *Hhip*^+*/-*^ and/or *Hhip* conditional knockout mice.

In summary, we linked the metabolic reprogramming to the function of genetic determinants in remodeling airways during COPD pathogenesis. Though further direct evidence in vivo is still needed, our current data from mixed models including primary human normal and COPD-derived ASMC cultures as well as *Hhip*^+*/-*^ mice suggested HHIP protected the lung from airway remodeling possibly by repressing ASMC hyperproliferation associated with metabolic reprogramming towards aerobic glycolysis. Our results highlight a novel activity for HHIP in airway remodeling and provide important insights into the molecular mechanisms by which HHIP may protect lung from airway remodeling and eventually lung function reduction during COPD pathogenesis, which may ultimately shed light on novel potential therapeutic approaches to limit airway remodeling in COPD pathogenesis.

## Supplementary Information

Supplementary Information 1.

Supplementary Information 2.

Supplementary Information 3.

Supplementary Information 4.
