# Supplementary material for: Hedgehog interacting protein (HHIP) represses airway remodeling and metabolic reprogramming in COPD-derived airway smooth muscle cells
Source: Sci Rep. 2021 Apr 27;11:9074. doi: 10.1038/s41598-021-88434-x (PMC8079715; doi:10.1038/s41598-021-88434-x)
Supplement: Supplementary file 1 — Supplementary Information 1. [file 41598_2021_88434_MOESM1_ESM.docx]

**Supplemental Table 1. Clinical Characteristics of patients for primary ASMCs used in this study**

| Group | Material (Lonza) | Batch# | Age | Gender | Alcohol | Smoking |
| --- | --- | --- | --- | --- | --- | --- |
| Healthy donnors  (N=4) | CC-2576 | 0000538849 | 59 Y | M | Y | Y |
|  | CC-2576 | 0000540803 | 49 Y | F | Y | Y |
|  | CC-2576 | 0000596065 | 52 Y | F | N/A | N/A |
|  | CC-2576 | 0000581076 | 57Y | M | N/A | N/A |
| COPD  (N=4) | 00195274 | 0000214249 | 49 Y | M | Y | Y |
|  | 00195274 | 0F3412 | 44 Y | M | N | Y |
|  | 00195274 | 0000178164 | 48 Y | F | Y | Y |
|  | 00195274 | 0F3348 | 48 Y | F | N/A | N/A |

N/A, not available.

Table 2. Characteristics of Women Who Later Had Preeclampsia and Controls at Enrollment in the Assisted Reproductive Cohort Study and Characteristics of Their Infants

| **Characteristic** | **Women with Preeclampsia (N=60)** | **Controls**  **(N=58)** | **P Value** |
| --- | --- | --- | --- |
| Age—yr  Height— cm  Weight—kg  Body-mass index  Systolic blood pressure—mmHg  Diastolic blood pressure—mmHg  Primigravida—no. (%)  Gestational age at enrollment—wk  Gestational age at delivery—wk  Current smoker—no. (%)  Infant’s birth weight—g  Delivery at<37 wk—no. (%)  Small-for-gestational-age infant  (<10^th^ percentile) —no. (%)  Frozen embryo transfer —no. (%)  Fresh embryo transfer —no. (%)  Singleton pregnancy rate—no. (%) |  |  |  |
| *Plus-minus values are mean ± SD. P values are given only for significant  differences  (此部分参考NEJM Reference 1 table 1) | | | |

Table 3. Characteristics of Preeclampsia Patients and Controls for Maternal serum BMP2 Level Measurements

| **Characteristic** | **Women with Preeclampsia (N=35)** | **Controls**  **(N=50)** | **P Value** |
| --- | --- | --- | --- |
| Age—yr  Height— cm  Weight—kg  Body-mass index  Systolic blood pressure—mmHg  Diastolic blood pressure—mmHg  Primigravida—no. (%)  Gestational age at enrollment—wk  Gestational age at delivery—wk  Current smoker—no. (%)  Infant’s birth weight—g  Delivery at<37 wk—no. (%)  Small-for-gestational-age infant  (<10^th^ percentile) —no. (%)  Frozen embryo transfer —no. (%)  Fresh embryo transfer —no. (%)  Singleton pregnancy rate—no. (%) |  |  |  |
| *Plus-minus values are mean ± SD. P values are given only for significant  differences | | | |
