# Supplementary figures and images for: Hedgehog interacting protein (HHIP) represses airway remodeling and metabolic reprogramming in COPD-derived airway smooth muscle cells
Source: Sci Rep. 2021 Apr 27;11:9074. doi: 10.1038/s41598-021-88434-x (PMC8079715; doi:10.1038/s41598-021-88434-x)

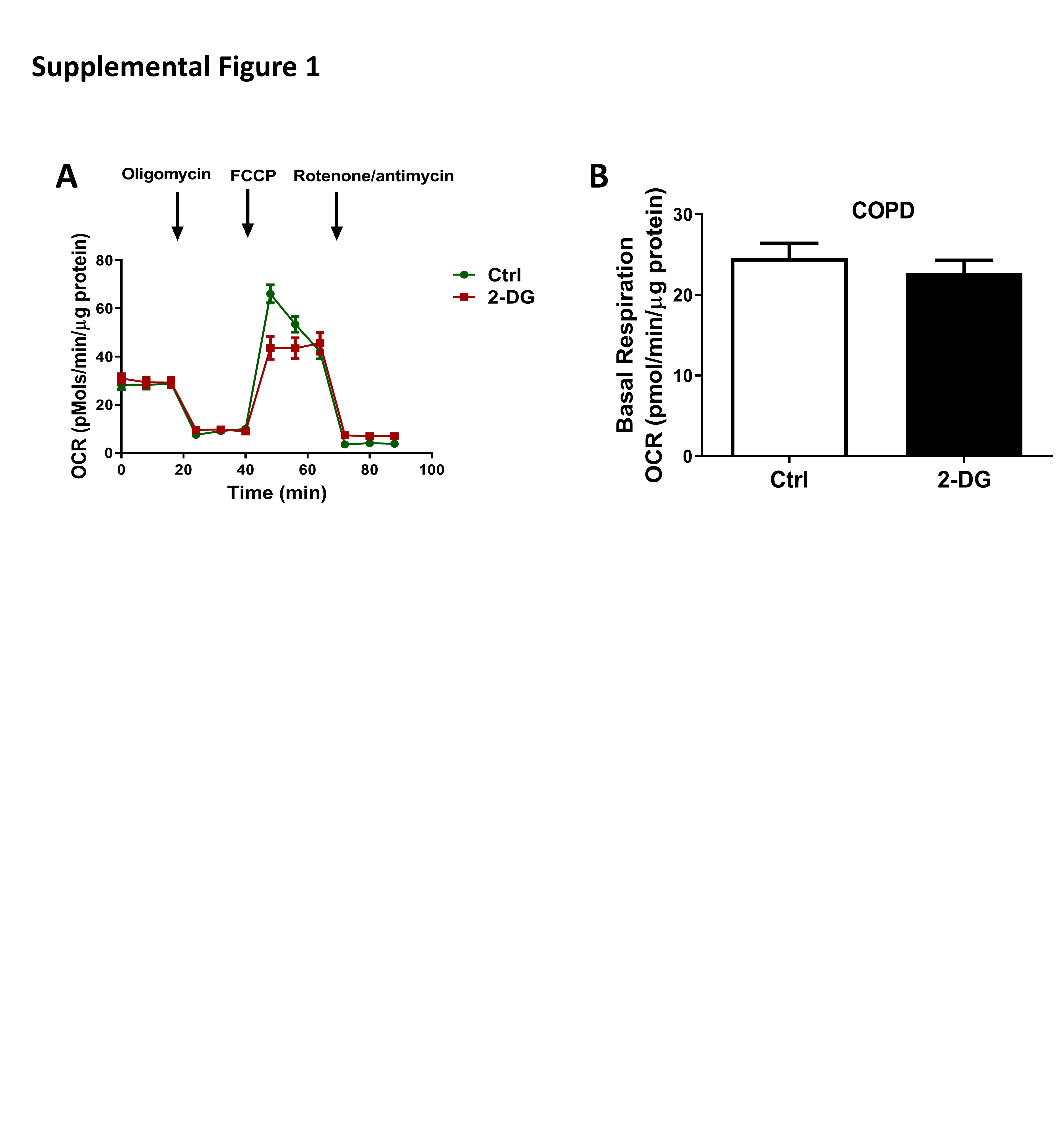

Supplement: Supplementary file 2 — Supplementary Information 2. [file 41598_2021_88434_MOESM2_ESM.tif]
