# Supplementary material for: Hedgehog interacting protein (HHIP) represses airway remodeling and metabolic reprogramming in COPD-derived airway smooth muscle cells
Source: Sci Rep. 2021 Apr 27;11:9074. doi: 10.1038/s41598-021-88434-x (PMC8079715; doi:10.1038/s41598-021-88434-x)
Supplement: Supplementary file 3 — Supplementary Information 3. [file 41598_2021_88434_MOESM3_ESM.docx]

**Supplemental Figure 1. 2-DG does not influence the oxidative phosphorylation rate of COPD-derived ASMCs.**

(A&B) The basal OCR was measured by Seahorse Mitostress assay in COPD-derived ASMCs with 2-DG treatment.

**Supplemental Table 1. Clinical Characteristics of patients for primary ASMCs used in this study**

The age, gender, alcohol, and smoking information of healthy donors and COPD patients (4 vs. 4) used for primary human ASMCs purchased from Lonza.
