# Supplementary material for: Hedgehog interacting protein (HHIP) represses airway remodeling and metabolic reprogramming in COPD-derived airway smooth muscle cells
Source: Sci Rep. 2021 Apr 27;11:9074. doi: 10.1038/s41598-021-88434-x (PMC8079715; doi:10.1038/s41598-021-88434-x)
Supplement: Supplementary file 4 — Supplementary Information 4. [file 41598_2021_88434_MOESM4_ESM.docx]

**Hedgehog interacting protein (HHIP) represses airway remodeling and metabolic reprogramming in COPD-derived airway smooth muscle cells**

Yan Li^1,2^*, Li Zhang^2, 3^, Francesca Polverino^4^, Feng Guo^2^, Yuan Hao^2^, Taotao Lao^5^, Shuang Xu^2^, Lijia Li^2^, Betty Pham^2^, Caroline A Owen^6^, Xiaobo Zhou^2,6^*
